# α-Methyltryptamine (α-MT) Metabolite Profiling in Human Hepatocyte Incubations and Postmortem Urine and Blood

**DOI:** 10.3390/metabo13010092

**Published:** 2023-01-06

**Authors:** Sara Malaca, Charline Bottinelli, Laurent Fanton, Nathalie Cartiser, Jeremy Carlier, Francesco Paolo Busardò

**Affiliations:** 1 Unit of Forensic Toxicology, Section of Legal Medicine, Department of Biomedical Sciences and Public Health, Marche Polytechnic University, Via Tronto 10/a, 60126 Ancona, Italy; 2 LAT LUMTOX Laboratory, 32 Rue du 35ème Régiment d’Aviation, 69500 Bron, France; 3 Department of Forensic Medicine, Edouard Herriot Hospital, Hospices Civils de Lyon, 5 Place d’Arsonval, 69003 Lyon, France

**Keywords:** tryptamine, α-MT, alpha-methyltryptamine, case report, metabolism, liquid chromatography-high-resolution tandem mass spectrometry, software-assisted data mining

## Abstract

α-MT is a hallucinogenic and stimulant tryptamine that was involved in several overdose fatalities in the United States and Europe. Analytical toxicology, and particularly the identification of metabolite biomarkers in biological samples, often is the only way to prove tryptamine use in clinical and forensic caseworks. We aimed to identify optimal α-MT metabolite biomarkers of consumption in humans. We identified α-MT metabolites in 10-donor-pooled human hepatocyte incubations and postmortem urine and blood from an α-MT overdose case using in silico metabolite predictions, liquid chromatography high-resolution-tandem mass spectrometry (LC-HRMS/MS), and software-assisted data mining. Nine metabolites were identified in vitro and eight additional metabolites were found in urine; five metabolites were found in blood. Metabolic transformations were hydroxylation, *O*-sulfation, *O*-glucuronidation, *N*-glucuronidation, and *N*-acetylation, consistent with the metabolism of structural analogues. The findings in hepatocyte incubations and postmortem samples were consistent, proving the in vitro model suitability. We suggest α-MT, hydroxy-α-MT glucuronide, and two hydroxy-α-MT sulfates as biomarkers of α-MT use in non-hydrolyzed urine; we suggest α-MT, two hydroxy-α-MT sulfates and *N*-acetyl-α-MT as biomarkers of α-MT use in blood. Further studies on α-MT clinical and forensic caseworks with different doses and routes of administration are necessary to better explore α-MT metabolism.

## 1. Introduction

Psychoactive tryptamines are indolealkylamines whose effects are mainly mediated by the serotonin (5-Hydroxy-*N*,*N*-tryptamine, 5-HT) receptors [1]. Naturally occurring tryptamines such as *N*,*N*-dimethyltryptamine (DMT) and psilocin (4-OH-*N*,*N*-dimethyltryptamine) have a long history of consumption in traditional medicine and magico-religious ceremonies, and are currently widely used as hallucinogens or entheogens [2,3]. Beside the risks of accidental injury inherent to hallucinogen use, tryptamines can induce a potentially fatal serotonin syndrome, and several analogues are therefore controlled under the United Nations 1971 Convention on Psychotropic Substances (Schedule I) [1,4]. In recent years, potent synthetic analogues have been used to circumvent scheduling laws and analytical detection; 57 analogues are currently monitored by the European Union Early Warning System of the European Monitoring Center for Drugs and Drug Addiction (EMCDDA) [5]. In the last few years, however, psychedelic tryptamines drew attention from researchers and media due to their tremendous therapeutic potential for treating common medical conditions such as migraine, post-traumatic stress disorder, depression, and drug addiction [6,7,8]. As such, the manufacturing of new synthetic analogues, the availability and the consumption of natural and synthetic tryptamines, and the occurrence of clinical and forensic cases involving these tryptamines are expected to rise. Law enforcement agencies and toxicology laboratories need to be prepared to cope with this upcoming challenge.

α-Methyltryptamine (α-MT) is a synthetic tryptamine available as a white and crystalline powder packaged in vials or capsules or pressed into tablets. It was initially developed in the Soviet Union in the 1960s as an antidepressant due to its monoamine oxidase inhibitor activity, but it was commercially unsuccessful [9]. Recreational use, however, has gained popularity in the 2000s due to prolonged psychedelic effects such as visual hallucinations and euphoria [10]. However, anxiety and depression are often reported the day after use [11], and high blood pressure, tachycardia, hyper-vigilance, mydriasis, tremor, delayed response time, restlessness, and exaggerated startle reaction can be observed in acute intoxication cases [10,12]. α-MT was involved in several fatalities in the United States, Great Britain, Sweden, Norway, and Japan [13]. Boland et al. reported two α-MT-overdose deaths, with a postmortem peripheral blood concentration of 2.0 µg/mL in one case, and an antemortem serum concentration of 1.5 µg/mL in the other case [14]. Although not scheduled under the United Nations 1971 Convention on Psychotropic Substances, it is controlled in several countries such as the United States (since 2003), Germany, Spain, and Austria [9,15].

Tryptamines induce non-specific effects, and the consumption of specific molecules can only be determined through the analysis of biological samples, such as blood and urine, in forensic and clinical settings. However, they can also be active at low doses and quickly metabolized, making it challenging for drug detection. For these reasons, targeting specific metabolite biomarkers is often preferred to improve detection and prove consumption [1]. α-MT metabolism was studied in vitro with rat liver microsomes [16] and in vivo with rat urine [16,17]. Incubation with rat liver microsomes produced 3-indolyacetone through oxidative deamination, 6-hydroxy-α-MT, and 6-hydroxy-3-indolyacetone, as identified by paper chromatography and color reactions; further glucuronide conjugates were also detected in rat urine [16]. More recently, Kanamori et al. identified four hydroxy-α-MT metabolites (1′-, 6-, and 7-hydroxy and 2-oxo) in rat urine by gas chromatography-mass spectrometry (GC-MS) after glucuronide/arylsulfate hydrolysis and derivatization; 4-, 5-, 3′-, and *N*- hydroxy-α-MT were not detected [17]. In the two studies, only specific metabolites were targeted, and the detection might not have been suitable due to lack of sensitivity [16] or potential thermosensitivity [17]. More importantly, whether in vitro or in vivo, there is currently no data on α-MT metabolism in humans.

To identify the most relevant biomarkers of α-MT consumption in clinical and forensic caseworks, we assessed α-MT human metabolism with: (1) in silico metabolite predictions to assist sample analysis and data mining, (2) in vitro incubations with primary human hepatocytes to simulate phase I and phase II metabolism in conditions similar to in vivo [18,19,20,21,22,23,24,25], and (3) the analysis of postmortem samples from an authentic overdose death to verify the in vitro results. Incubates and samples were analyzed by liquid chromatography-high-resolution tandem mass spectrometry (LC-HRMS/MS) in positive- and negative-ionization modes and software-assisted data mining for an all-inclusive screening of α-MT metabolites.

## 2. Materials and Methods

### 2.1. In Silico Metabolite Prediction

BioTransformer freeware (v.3.0) was employed to predict α-MT first- and second-generation metabolites in humans [26,27]. The metabolite list was generated using α-MT simplified molecular-input line-entry system (SMILES) string with the “Human and human gut microbial transformation (All human)” option and “combined” CYP450 mode. The accurate mass of predicted metabolites were compiled in an inclusion list for LC-HRMS/MS analysis to prioritize the fragmentation of specific targets (Supplementary Table S1). All predicted metabolic reactions and combinations were included in the list of possible transformations for data mining.

### 2.2. Chemicals and Reagents

LC-MS grade methanol, acetonitrile, water, and formic acid (FA) were bought from Carlo Erba (Cornaredo, Italy). LC-MS grade acetic acid and ammonium acetate were acquired by Levanquimica (Bari, Italy). α-MT and diclofenac analytical standards were purchased from Toronto Research Chemicals (North York, Canada) and Sigma Aldrich (Milan, Italy), respectively. Standards were solubilized in LC-MS grade methanol to 1 mg/mL stock solutions and stored at −20 °C until analysis. Ten-donor-pooled cryopreserved human hepatocytes and thawing medium (TM) were obtained from Lonza (Basel, Switzerland). Supplemented Williams’ Medium E (sWME) was prepared with 2 mmol/L *l*-glutamine and 20 mmol/L HEPES (2-[4-(2-hydroxyethyl)-1-piperazinyl]ethanesulfonic acid) in Williams’ Medium E from Sigma Aldrich, prior to the analysis. β-Glucuronidase (50 units/µL) from limpets (*Patella vulgata* L.) was purchased from Sigma Aldrich.

### 2.3. Hepatocyte Incubations

Incubations were carried out as previously described with a few minor modifications [28]. Hepatocytes were thawed at 37 °C and gently mixed with 50 mL TM in a 50 mL polypropylene conical tube at 37 °C. After centrifugation at 50× *g* for 5 min, the cells were washed with 50 mL sWME at 37 °C then resuspended in 2 mL sWME after centrifugation in the same conditions. Hepatocyte viability was 87%, as evaluated with the trypan blue exclusion test, and sWME volume adjusted to 1.65 × 10^6^ viable cells/mL. In a sterile 24-well culture plate, 200 μL hepatocyte suspension was gently mixed with 200 μL α-MT at 20 μmol/L in sWME, and the plate was immediately incubated at 37 °C. Metabolic reactions were then interrupted with 400 µL ice-cold acetonitrile after 0 or 3 h incubation. The incubates were centrifuged at 15,000× *g* for 5 min then stored at −80 °C. Negative controls, i.e., hepatocytes in sWME without α-MT and α-MT in sWME without hepatocytes, were incubated for 0 or 3 h under the same conditions. Diclofenac was also incubated under the same conditions to ensure proper metabolic activity.

### 2.4. Authentic Samples

Femoral/peripheral blood, cardiac blood, urine, and bile were collected from a fatal case of acute cardiac circulatory collapse secondary to a polydrug intoxication involving α-MT. The subject was a 35-year-old Caucasian male weighing 50 kg and 178 cm tall. Samples were stored at −20 °C until analysis and between tests.

α-MT concentrations were 4.7 µg/mL in peripheral and cardiac blood and higher than 5.0 µg/mL, the upper limit of quantification, in urine and bile. Additionally, in-house toxicology screenings and subsequent confirmation methods by gas or liquid chromatography-tandem mass spectrometry (GC- or LC-MS/MS, respectively) revealed co-exposure to ephedrine, diazepam, and benzofuran derivative 5-MAPB. 5-MAPB concentrations were 101, 27.4, 4170, and 1450 ng/mL in peripheral blood, cardiac blood, urine, and bile, respectively; concentrations of major metabolite 5-APB were 9.33, 5.74, 262, and 43.6 ng/mL, respectively.

α-MT metabolite profiling was conducted in peripheral blood and urine, as described below.

### 2.5. Sample Preparation

#### 2.5.1. Incubates

After thawing at room temperature and mixing, incubates were centrifuged at 15,000× *g* for 5 min. A volume of 100 μL supernatant was vortexed with 100 μL acetonitrile and centrifuged at 15,000× *g* for 10 min. The supernatants were dried under nitrogen at 37 °C in conical glass tubes, and the residues were reconstituted with 100 μL 0.1% FA in water. After centrifugation at 15,000× *g* for 10 min, the supernatants were transferred into glass inserts in LC autosampler vials with a glass insert. Controls were prepared with the samples under the same conditions without β-glucuronidase to rule out non-enzymatic hydrolysis.

#### 2.5.2. Urine and Blood

Samples were thawed at room temperature, and 100 µL blood or 50 µL urine was mixed with 200, or 100 µL acetonitrile, respectively, and centrifuged at 15,000× *g* for 10 min. The supernatants were evaporated to dryness under nitrogen at 37 °C, and the residues were reconstituted with 100 µL 0.1% FA in water. After centrifugation at 15,000× *g* for 10 min, the supernatants were transferred in autosampler vials with a glass insert.

Additionally, to study phase II metabolites, urine was prepared after glucuronide hydrolysis. A volume of 50 µL sample was mixed with 50 µL water, 10 µL 10 mol/L ammonium acetate, pH 5.0, and 100 µL β-glucuronidase in conical glass tubes and incubated at 37 °C for 90 min. After hydrolysis, the samples were vortexed with 400 µL ice-cold acetonitrile and dried under nitrogen at 37 °C in a conical glass tube. The residues were reconstituted with 100 μL 0.1% FA in water and centrifuged at 15,000× *g* for 10 min. The supernatants were transferred into LC autosampler vials with a glass insert. Controls were prepared with the samples under the same conditions without β-glucuronidase to rule out non-enzymatic hydrolysis.

### 2.6. Instrumental

LC-HRMS/MS analysis was performed with a Dionex UltiMate 3000 chromato-graphic system coupled with a Thermo Scientific (Waltham, MA, USA) Q Exactive mass spectrometer equipped with a heated electrospray ionization (HESI) source. Each sample was injected once in positive- and once in negative-ionization mode (15 µL).

#### 2.6.1. Liquid Chromatography

Separation was performed with a Kinetex Biphenyl column (150 × 2.1 mm, 2.6 μm) from Phenomenex (Castel Maggiore, Italy) with a mobile phase gradient composed of 0.1% FA in water (MPA) and 0.1% FA in acetonitrile (MPB) at 37 ± 1 °C. Run time was 21 min with a 0.4 mL/min flow rate. The gradient started with 2% MPB for 2 min; MPB was increased to 15% within 10 min then 95% within 2 min and held for 4 min before returning to initial conditions within 0.1 min; re-equilibration time was 2.9 min. Autosampler temperature was 10 ± 1 °C.

#### 2.6.2. High-Resolution Tandem Mass Spectrometry

HESI source parameters were: sheath gas flow rate, 50 u.a.; auxiliary gas flow rate, 10 u.a.; spray voltage, ±3.5 kV; capillary temperature, 300 °C; auxiliary gas heater temperature, 100 °C; S-lens radio frequency, 50 u.a.; sweep gas was not applied. The orbitrap was calibrated prior to analysis, and a lock mass list composed of previously identified contaminants was used during injections for better accuracy (phthalates with *m/z* 279.1591, 301.1410, and 391.2843 in positive-ionization mode and trifluoro-acetic acid with *m/z* 248.9604 in negative-ionization mode [29]).

Data were acquired from 1 to 18 min in full scan HRMS (FullMS)/data-dependent MS/MS (ddMS^2^) mode. The FullMS acquisition range was *m/z* 150–520 with a resolution of 70,000 at full width at half maximum (FWHM) at *m/z* 200; the automatic gain control (AGC) target was 10^6^ and the maximum injection time (IT) 200 ms. Up to five ddMS^2^ scans were triggered, with a dynamic exclusion of 2.0 s and an intensity threshold of 10^4^, for each FullMS scan depending on a priority inclusion list of putative metabolites based on in silico predictions and the metabolic fate of α-MT analogues [16,17,30,31,32,33,34,35,36,37] (Supplementary Table S1). ddMS^2^ isolation window was *m/z* 1.2 with a resolution of 17,500 and the normalized collision energy (NCE) was 20, 90, and 110 a.u.; AGC target was 2 × 10^5^ and maximum IT was 64 ms. Additionally, background *m/z* values with high intensity were assessed during the injection of blank controls and compiled in an exclusion list in positive and negative-ionization modes.

### 2.7. Data Mining

LC-HRMS data were processed with Thermo Scientific Compound Discoverer (v.3.1.1.12), using a partially automated targeted/untargeted approach, as previously described with minor modifications [28]. Briefly, the ions detected in HRMS were compared to a list of theoretical metabolites based on in silico predictions, the metabolic fate of α-MT analogues, and postulation, and generated using combinations of the following transformations: desaturation (2H > ø), dihydrodiol formation (ø > 2O 2H), ketone formation (2H > O), oxidation (ø > O), oxidative deamination to alcohol (2H N > H O), oxidative deamination to ketone (3H N > O), reduction (ø > 2H); acetylation (H > 2C 3H O), glucuronidation (H > 6C 9H 6O), glycine conjugation (H O > 2C 4H N 2O), glutathionylation (ø > 10C 17H 3N 6O S), methylation (H > C 3H), and sulfation (H > H 3O S); in-source amine loss, abundant for α-MT, was added to the phase I transformation list (3H N > ø); the maximum number of dealkylations was 2, the maximum number of phase II reactions was 2, and the maximum number of transformations was 4. LC-HRMS intensity threshold was 5 × 10^3^ and HRMS mass tolerance 5 ppm. The HRMS/MS spectra and theoretical elemental composition of the ions were compared to mzCloud (Drugs of Abuse/Illegal Drugs, Endogenous Metabolites, and Natural Products/Medicines libraries), ChemSpider (Cayman Chemical and DrugBank libraries), and HighResNPS online databases: intensity threshold, 10^5^; HRMS mass tolerance, 5 ppm; HRMS/MS mass tolerance; 10 ppm. The chromatographic peaks detected in controls with a similar or higher intensity than that of the peaks detected in the samples were disregarded. Molecules with a signal intensity lower than 0.5% of that of the metabolite with the most intense signal were also disregarded.

## 3. Results

### 3.1. In Silico Metabolite Predictions

Human metabolite predictions are presented in Supplementary Table S2. A total of ten first-generation (P1–P10) and 67 s-generation metabolites (P11–P79, including two replicates, i.e., P32/PP41 and P61/P67) were predicted. First-generation metabolites were produced by hydroxylation, *N*-oxidation, terminal desaturation, and oxidative deamination, and second-generation metabolites also included desaturation to ketone, ketoreduction, *O*-glucuronidation, and *O*-sulfation; glucuronidation and sulfation were the only phase II reactions. To assist in LC-HRMS/MS analysis, all the predicted metabolites were included in an inclusion list (Supplementary Table S1). To support automatic data mining, all predicted metabolic transformations were included in the list of potential reactions.

### 3.2. α-MT HRMS/MS Fragmentation Pattern

**Figure 1:**
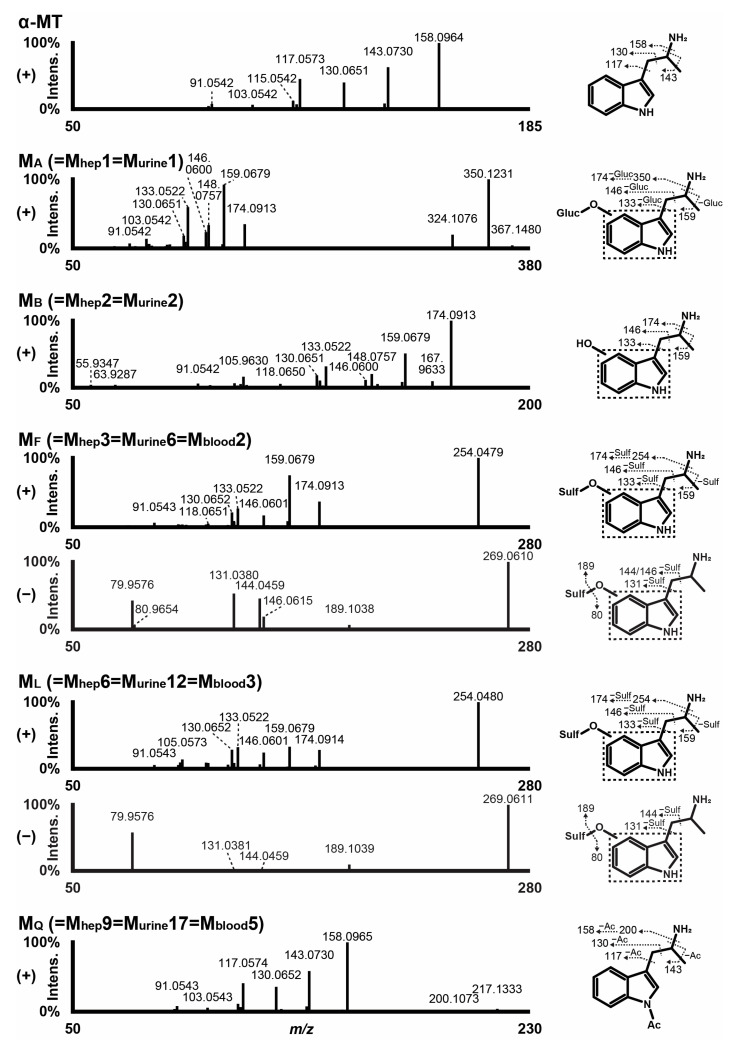
Fragmentation pattern of α-MT and major metabolites in liquid chromatography-high-resolution tandem mass spectrometry. We suggest M_A_, M_F_, and M_L_ as biomarkers of α-MT use in non-hydrolyzed urine, α-MT and M_B_ in hydrolyzed urine, and α-MT, M_F_, M_L_, and M_Q_ in blood. (+), positive-ionization mode; (−), negative-ionization mode; Ac, acetyl; Gluc, glucuronide; Sulf, sulfate; dotted box, Markush structure.

α-MT main site of ionization was the primary amine of the alkyl side chain in positive-ionization mode, major fragment *m/z* 158.0964 (C_11_H_12_N^+^) being yielded through α-cleavage (amine loss). Further β-cleavage produced fragments *m/z* 143.0730 (C_10_H_9_N^+^) and 130.0651 (C_9_H_8_N^+^). Fragments *m/z* 117.0573 (C_8_H_7_N^+^) and 115.0542 (C_9_H_7_^+^) were typical of the indole group (Figure 1). Despite the optimization of the ion source settings, α-MT signal intensity was low compared to that of other tryptamines in similar LC-HRMS conditions [37].

This can be partly explained by a considerable in-source amine loss, as *m/z* 158.0964 intensity was approximately 3.3 times higher than that of parent. α-MT did not produce a signal in negative-ionization mode in the experimental conditions.

### 3.3. α-MT Metabolites in Human Hepatocyte Incubations

Diclofenac metabolization to 4′-hydroxydiclofenac and acyl-β-D-glucuronide diclofenac after 3 h incubation as a control alongside α-MT in the same conditions indicated that α-MT in vitro metabolism occurred properly. α-MT signal intensity was 6.0 × 10^7^ and 4.5 × 10^7^ in human hepatocyte incubations for 0 and 3 h, respectively. Nine metabolites were identified after 3 h incubation and listed from M_hep_1 to M_hep_9 by ascending retention time. Metabolic reactions were hydroxylation (M_hep_2 and M_hep_4) and further *O*-sulfation (M_hep_3 and M_hep_6) or *O*-glucuronidation (M_hep_1 and M_hep_5), *N*-acetylation (M_hep_9), and combinations (M_hep_7 and M_hep_8).

The fragmentation pattern of α-MT major metabolites in positive- and negative-ionization mode (when applicable) is reported in Figure 1, and the fragmentation pattern of all metabolites is reported in Supplementary Figure S1. The elemental composition, retention time, accurate mass of molecular ion, and LC-HRMS peak area of α-MT and metabolites in positive- and negative-ionization mode after 3 h incubation with hepatocytes are reported in Table 1.

#### 3.3.1. α-MT Hydroxylation and Further *O*-Sulfation or *O*-Glucuronidation

M_hep_2 and M_hep_4 eluted at 4.65 and 6.15 min of the LC run time, respectively, with a +15.9949 Da mass shift from parent, indicating oxidation (+O). Both molecules presented a fragmentation pattern similar to that of α-MT in positive-ionization mode, fragments *m/z* 174.0913, 159.0679, 146.0600, and 133.0522 carrying the metabolic transformation (*m/z* 158.0964, 148.0730, 130.0651, and 117.0573, respectively, from parent +O) and indicating a hydroxylation at the indole core. The exact position of the hydroxyl group at M_hep_2 and M_hep_4 indole core cannot be ascertained in the present analytical conditions. M_hep_2 and M_hep_4 were fragmented along with *m/z* 190.9795 and other minor ions detected during the whole LC separation, producing interferences such as *m/z* 105.9630, 149.9527, and 167.9633.

M_hep_3 and M_hep_6 eluted at 5.40 and 7.48 min of the chromatographic gradient, respectively, with a +95.9516 Da mass shift from parent, indicating oxidation (+O) and sulfation (+SO_3_). Interestingly, sulfation delayed retention times when compared to M_hep_2 and M_hep_4, as observed previously with other tryptamines in similar LC conditions [37]. In positive-ionization mode, amine loss (*m/z* 254.0480) and fragments *m/z* 174.0914, 159.0679, 146.0601, and 133.0522, also observed in hydroxy-α-MT metabolites, pointed towards *O*-sulfation at the indole core. M_hep_3 and M_hep_6 also produced a more intense signal in negative-ionization mode due to the sulfate proneness to form an anion. After negative ionization, sulfate cleavage produced fragments *m/z* 79.9576 and 189.1039, and fragments *m/z* 144.0459 and 131.0381 confirmed that the metabolic reactions occurred at the indole core.

M_hep_1 and M_hep_5 eluted at 3.06 and 6.35 min, respectively, with a +192.0270 Da mass shift from α-MT, indicating oxidation (+O) and glucuronidation (+C_6_H_8_O_6_). In positive-ionization mode, amine loss (*m/z* 350.1231) and fragments *m/z* 174.0913, 159.0679, 146.0600, and 133.0522 pointed towards *O*-glucuronidation at the indole core in both molecules.

#### 3.3.2. α-MT *N*-Acetylation and Combinations

M_hep_9 was highly retained and eluted at 14.07 min of the LC gradient with a +42.0105 Da mass shift from α-MT, indicating *N*-acetylation (+C_2_H_2_O). M_hep_9 fragmentation pattern after positive ionization contained ion *m/z* 200.1073 through amine loss, indicating that the reaction did not occur at the primary amine of the molecule but rather at the secondary amine of the indole core; fragments *m/z* 158.0965, 143.0730, 130.0652, and 117.0574 were also detected in α-MT HRMS/MS spectrum.

M_hep_7 and M_hep_8 eluted at 9.48 and 9.62 min, respectively. Based on M_hep_7 retention time and HRMS/MS in positive- and negative-ionization modes, it was produced by hydroxylation (+O) and subsequent *O*-sulfation (+SO_3_) and *N*-acetylation (+C_2_H_2_O) at the indole core. Similarly, M_hep_8 was the result of hydroxylation (+O) and subsequent *O*-glucuronidation (+C_6_H_8_O_6_) and *N*-acetylation (+C_2_H_2_O) at the indole core.

### 3.4. α-MT Metabolites in Postmortem Urine

Seventeen metabolites were identified in urine and listed from M_urine_1 to M_urine_17 by ascending retention time. α-MT signal was 10 times higher than that of the metabolite with the most intense signal (M_urine_6). All the metabolites identified after 3 h incubation with human hepatocytes were detected in urine: M_hep_1 = M_urine_1, M_hep_2 = M_urine_2, M_hep_3 = M_urine_6, M_hep_4 = M_urine_8, M_hep_5 = M_urine_9, M_hep_6 = M_urine_12, M_hep_7 = M_urine_13, M_hep_8 = M_urine_14, and M_hep_9 = M_urine_17. Eight additional metabolites were identified with hydroxylation and further *O*-sulfation (M_urine_5) or *O*-glucuronidation (M_urine_3, M_urine_4, and M_urine_7), *N*-glucuronidation (M_urine_10, M_urine_11, and M_urine_15), and combination (M_urine_16). Besides M_urine_7, whose signal was approximately reduced by a factor of 10, none of the *O*-glucuronides (M_urine_1, M_urine_3, M_urine_4, M_urine_9, and M_urine_14) were detected after hydrolysis. In contrast, the signal of hydroxy- α-MT metabolites M_urine_2 and M_urine_8 increased 34 and 25 times, respectively. Considering their structure, the metabolites identified in postmortem samples could not be produced by the other substances detected during toxicology analyses, including the benzofuran derivative 5-MAPB.

The fragmentation pattern of α-MT major metabolites in positive- and negative-ionization mode (when applicable) is reported in Figure 1, and the fragmentation pattern of all metabolites is reported in Supplementary Figure S1. The elemental composition, retention time, accurate mass of molecular ion, and LC-HRMS peak area of α-MT and metabolites in positive- and negative-ionization mode in urine with and without hydrolysis are reported in Table 2.

#### 3.4.1. α-MT Hydroxylation and *O*-Sulfation or *O*-Glucuronidation

M_urine_1 (M_hep_1) and M_urine_9 (M_hep_5) susceptibility to β-glucuronidase hydrolysis confirmed the formation of *O*-glucuronides.

M_urine_3, M_urine_4, and M_urine_7 eluted at 4.81, 4.88, and 5.98 min of the LC gradient, respectively, with a +192.0268 Da mass shift from α-MT, indicating oxidation (+O) and glucuronidation (+C_6_H_8_O_6_); M_urine_3 and M_urine_4 partially coeluted. M_urine_3, M_urine_4, and M_urine_7 fragmentation pattern was similar to that of previously identified *O*-glucuronides, with fragments *m/z* 350.1239, 174.0914, 159.0678, 146.0599, and 133.0522 in positive-ionization modes, indicating *O*-glucuronidation at the indole core. In M_urine_3 and M_urine_4, fragment relative intensity differed from that of previously identified *O*-glucuronides, the amine loss (*m/z* 350.1239) being substantially less intense.

M_urine_5 eluted at 5.08 min, with a +95.9515 Da mass shift from α-MT and a fragmentation pattern similar to that of previously identified *O*-sulfates in positive- and negative-ionization modes, indicating *O*-sulfation at the indole core (+O +SO_3_).

#### 3.4.2. α-MT *N*-Glucuronidation and Combination

M_urine_10 and M_urine_11 partially co-eluted at 7.06 and 7.15 min of the LC gradient, respectively, with a +176.0317 Da mass shift from α-MT, indicating *N*-glucuronidation (+C_6_H_8_O_6_). In positive-ionization mode, M_urine_10 and M_urine_11 produced fragment *m/z* 334.1281 through amine loss and fragments *m/z* 158.0964, 143.0730, 130.0651, and 117.0573, also observed in parent, indicating *N*-glucuronidation at the indole core. M_urine_10 and M_urine_11 were not affected by the enzymatic hydrolysis, which indeed catalyzes the breakdown of *O*-glucuronides.

M_urine_15 eluted much later, at 10.55 min of the gradient, also with a +176.0320 Da mass shift from parent, indicating *N*-glucuronidation (+C_6_H_8_O_6_). However, in addition to fragments *m/z* 158.0965, 143.0730, 130.0651, and 117.0573, also observed in α-MT, fragment *m/z* 220.0814, produced by β-cleavage at the amine of the alkyl side chain, carried the transformation and indicated *N*-glucuronidation at the alkyl side chain. Fragment *m/z* 334.1281 was not detected confirming the position of the transformation. M_urine_15 was not affected by the enzymatic hydrolysis.

Considering M_urine_16 retention time (12.98 min), and HRMS/MS in positive-ionization mode, it was produced by *N*-glucuronidation (+C_6_H_8_O_6_) and *N*-acetylation (+C_2_H_2_O) at the indole core.

### 3.5. α-MT Metabolites in Postmortem Blood

Five metabolites were identified in blood and listed from M_blood_1 to M_blood_5 by ascending retention time. α-MT signal was 10 times higher than that of the metabolite with the most intense signal (M_blood_2). All the metabolites identified in urine were detected in blood: M_blood_1 = M_urine_5, M_blood_2 = M_urine_6, M_blood_3 = M_urine_12, M_blood_4 = M_urine_13, and M_blood_5 = M_urine_17.

The fragmentation pattern of α-MT major metabolites in positive- and negative-ionization mode (when applicable) is reported in Figure 1, and the fragmentation pattern of all metabolites is reported in Supplementary Figure S1. The elemental composition, retention time, accurate mass of molecular ion, and LC-HRMS peak area of α-MT and metabolites in positive- and negative-ionization mode in blood are reported in Table 3.

## 4. Discussion

### 4.1. General Analytical Considerations

The structure elucidation of α-MT metabolites is challenging for several reasons: (1) α-MT has a low molecular mass (174.2 g/mol), and α-MT and metabolites therefore produce poor HRMS/MS spectra and are often interfered with by ions with the same elemental composition in HRMS and HRMS/MS; (2) α-MT and metabolites produce a low signal intensity in HRMS, which can be partly explained by a substantial in-source fragmentation due to amine loss; and (3) the detection of α-MT metabolites after in-source fragmentation may prompt misinterpretation of the metabolic transformations. To limit isomer co-elution and the occurrence of interferences, the LC gradient was developed using a 15-cm-long analytical column with a biphenyl stationary phase (π-π interaction with the indole group of the molecules) and a particularly slow increase of the organic phase percentage. To limit in-source fragmentation, the auxiliary gas heater temperature was maintained to the minimal recommended value, and amine loss was added to the list of potential reactions for data mining to avoid missing any potential metabolite due to a lack of sensitivity.

In silico metabolite predictions helped in compiling LC-HRMS/MS inclusion and exclusion lists and implementing the transformation list for data mining with Compound Discoverer. Except for *N*-acetylation, all reactions were predicted; predicted reactions such as hydroxylation/oxidation at the alkyl chain, oxidative deamination, and terminal desaturation were not detected. In silico metabolite predictions alone are not sufficient to accurately anticipate in vivo metabolism, warranting the use of in vitro models and the analysis of authentic samples. Additionally, they should be considered carefully when analyzing sample results to avoid biased interpretation [27].

### 4.2. In Vitro Versus Postmortem Metabolites

**Figure 2:**
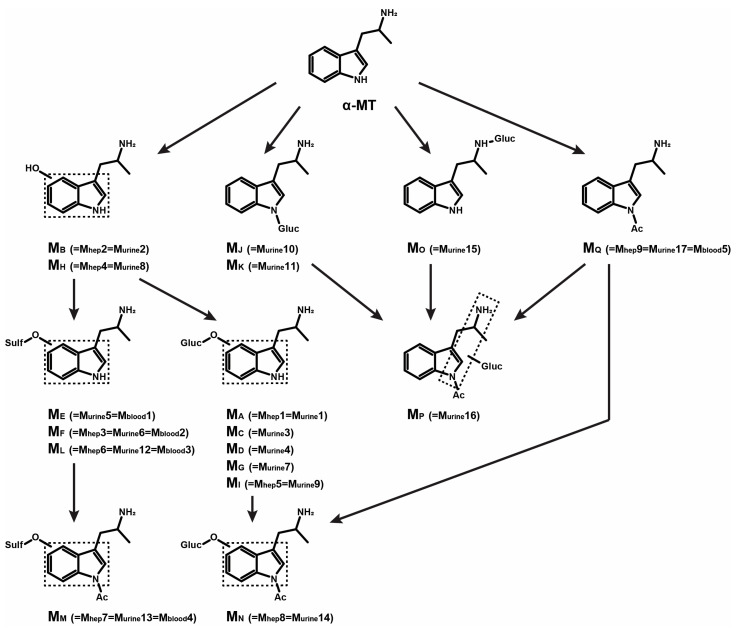
α-MT suggested metabolic fate in humans. Ac, acetyl; Gluc, glucuronide; Sulf, sulfate; dotted box, Markush structure.

Table 4 summarizes in vitro and postmortem findings, renaming the metabolites from M_A_ to M_Q_ for better clarity, the elemental composition, retention time in urine, and theoretical accurate mass of molecular ion of α-MT and metabolites in positive- and negative-ionization modes. α-MT metabolic fate in humans is suggested in Figure 2.

All nine metabolites identified after 3 h incubation with human hepatocytes were detected in postmortem urine. Eight additional metabolites were identified in urine: M_C-E_, M_G_, M_J_, M_K_, M_O_, and M_P_. The additional metabolites were all minor, suggesting that the incubation with 10-donor-pooled human hepatocytes is a good model to predict α-MT human metabolism. However, the relative intensity of *O*-glucuronides and *O*-sulfates was much higher in urine than in vitro, which may be due to extrahepatic metabolism, and the relative intensity of *N*-acetyl metabolites were lower. Therefore, while M_B_ (hydroxy-α-MT), M_L_ (hydroxy-α-MT sulfate), and M_Q_ (*N*-acetyl-α-MT) were the most intense metabolites in hepatocyte incubations, M_A_ (hydroxy-α-MT glucuronide), M_F_ (hydroxy-α-MT sulfate), and M_L_ (hydroxy-α-MT sulfate) were preponderant in non-hydrolyzed urine, and M_B_ (hydroxy-α-MT), M_F_ (hydroxy-α-MT sulfate), and M_L_ (hydroxy-α-MT sulfate) were preponderant in urine after β-glucuronide hydrolysis.

Expectedly, fewer metabolites were identified in postmortem blood due to elimination. Glucuronides were quickly excreted and were not detected in blood. More interestingly, however, hydroxy-α-MT metabolites also were not detected, likely due to low intensity, considering the overall intensity of α-MT and metabolites. Consistent with in vitro and urinary results, M_F_ (hydroxy-α-MT sulfate), M_L_ (hydroxy-α-MT sulfate), and M_Q_ (*N*-acetyl-α-MT) were the most intense metabolites detected in blood.

Remarkably, α-MT signal was much more intense than that of the metabolites in postmortem samples, possibly because the individual died of overdose. However, it cannot be excluded that a significant proportion of α-MT is eliminated without alteration. α-MT metabolization was slow in hepatocyte incubations, considering α-MT signal intensity after 0 and 3 h (−25% difference), corroborating the latter statement. A slow metabolism could also explain α-MT prolonged psychedelic effects [10]. Postmortem samples were obtained from a single case of α-MT overdose, and α-MT metabolism may differ with the dose, the route of administration, the time of collection after intake, postmortem redistribution (when applicable), and interindividual genetic variations. Additionally, the stability of α-MT metabolites in postmortem samples after multiple freeze/thaw cycles is unknown. For these reasons, the analysis of other samples from authentic clinical and forensic caseworks are necessary to better understand α-MT pharmacokinetics.

### 4.3. Comparison to Analogues

Consistent with the present results, phase II transformations at the indole core are major pathways of tryptamine metabolism, either by direct glucuronidation or following hydroxylation or *O*-dealkylation [1,30,34,37]. *O*-Glucuronide is the main metabolite target of psilocin consumption in human urine [34], and *O*-glucuronide and *O*-sulfate are major 5-methoxy-*N*,*N*-diisopropyltryptamine (5-MeO-DiPT) metabolites in urine [30].

Indole hydroxylation was minor (before hydrolysis) in the present experiments, consistent with the metabolism of tryptamine analogues [1,30,34,36].

5-HT can undergo *N*-acetylation at the alkyl chain through the aralkylamine *N*-acetyltransferase, mainly expressed in the central nervous system, and the arylamine *N*-acetyltransferases, ubiquitously expressed, as a step of melatonin biosynthesis in vertebrates [38]; other 2-arylethylamines, such as tryptamine, 5-methoxytryptamine, and phenylethylamine, also are aralkylamine *N*-acetyltransferase substrates [39]. Interestingly, however, indole *N*-acetylation was not reported in the metabolic fate of structural analogues and was not predicted in silico, highlighting the necessity of an untargeted screening of LC-HRMS/MS data for metabolite identification studies. Comparison to reference standards, which are yet to be synthesized, is necessary to definitely confirm the position of the acetylation.

### 4.4. Comparison to α-MT Metabolism in Rats

Szara et al. identified 3-indolyacetone through oxidative deamination, 6-hydroxy-α-MT, 6-hydroxy-3-indolyacetone, and further *O*-glucuronide conjugates as α-MT metabolites in rats [16]. More recently, Kanamori et al. identified 1′-, 6-, and 7-hydroxy- and 2-oxo-α-MT also in rats [17]. Although these two studies only targeted specific metabolites, the overall results were different in humans. Most metabolites identified in humans were phase II metabolites, but glucuronides were minor in Szara et al.’s study [16], and phase II metabolism was not assessed by Kanamori et al., who performed β-glucuronidase/arylsulfatase hydrolysis prior to sample analysis [17]. Importantly, oxidative deamination, a major detoxication pathway of *N*,*N*-dimethyl tryptamines, was not detected in the present experiments, likely due to the methyl group protective effect [1]. Consistent with users’ reports, the lack of 3-indolyacetone suggests that α-MT does not require co-administration of a monoamine oxidase inhibitor to effectively induce psychedelic effects [10]. The differences between rat and human metabolism is not surprising due to inter-species discrepancies. Although metabolic studies in rats are a convenient model to predict human drug metabolism, studies in humans are necessary to confirm these preliminary results.

### 4.5. Recommended Biomarkers of Consumption

We suggest α-MT and major metabolites M_A_ (hydroxy-α-MT glucuronide), M_F_ (hydroxy-α-MT sulfate), and M_L_ (hydroxy-α-MT sulfate) as biomarkers of α-MT consumption in urine in clinical and forensic toxicology; M_F_ and M_L_ detectability is notably higher in negative-ionization mode. At present, due to the lack of analytical standards for the newly identified metabolites, hydrolysis is not recommended as there is no guarantee to completely cleave *O*-glucuronides and *O*-sulfates without proper optimization of the hydrolysis conditions. Additionally, the total signal of *O*-glucuronides without hydrolysis was more intense than that of hydroxy-α-MT metabolites with hydrolysis and targeting *O*-glucuronides seems therefore more rational. Nonetheless, in the case of urinary hydrolysis, we suggest α-MT and M_B_ (hydroxy-α-MT) as biomarkers of consumption.

We suggest α-MT and major metabolites M_F_ (hydroxy-α-MT sulfate), M_L_ (hydroxy-α-MT sulfate), and M_Q_ (*N*-acetyl-α-MT) as biomarkers of α-MT consumption in blood.

To the best of our knowledge, these metabolites were not identified in the metabolism of structural analogues and are specific to α-MT. It should be kept in mind, however, considering the novel psychoactive substance market dynamics, that new analogues potentially sharing metabolites with α-MT such as hydroxy-α-MT may emerge onto the illicit drug market in the future.

## 5. Conclusions

We studied α-MT metabolism in humans for the first time. We identified α-MT metabolites in human hepatocytes and postmortem urine and whole blood in an overdose casework using in silico metabolite predictions, LC-HRMS/MS analysis, and software-assisted data mining. Seventeen metabolites were identified in authentic samples with hydroxylation, *O*-sulfation, *O*-glucuronidation, *N*-glucuronidation, and *N*-acetylation; the transformations mainly occurred at the indole core of the molecule. We suggest α-MT, hydroxy-α-MT glucuronide M_A_, and hydroxy-α-MT sulfates M_F_ and M_L_ as biomarkers of α-MT use in non-hydrolyzed urine. We suggest α-MT, hydroxy-α-MT sulfates M_F_ and M_L_ and *N*-acetyl-α-MT M_Q_ as biomarkers of α-MT use in blood. The findings in postmortem samples were consistent with those observed in vitro, confirming the suitability of 10-donor-pooled human hepatocyte incubations as a model to predict tryptamine metabolism in humans. Tryptamines are prone to phase II conjugations, which are usually quickly excreted after formation. All nine metabolites identified in hepatocytes were indeed found in urine, eight additional minor metabolites being found in urine.

However, further studies on α-MT clinical and forensic caseworks with different doses and routes of administration are necessary to explore α-MT metabolism, particularly to understand parent detectability compared to that of its metabolites in authentic samples. Our results provide important data to orientate analytical standard manufacturers’ synthesis effort and will help toxicologists identify new cases to generate biological samples to refine the present results.

## Figures and Tables

**Table 1:** Metabolic transformation, elemental composition, retention time (RT), accurate mass of molecular ion in positive- and negative-ionization modes ([M+H]^+^ and [M−H]^−^, respectively) with mass error, and liquid chromatography–high-resolution mass spectrometry peak area of α-MT and metabolites after 3 h incubation with human hepatocytes.

ID	Metabolic Transformation	Elemental Composition	RT, min	*m/z* (Δppm): [M+H]^+^ [M−H]^−^	Peak Area [M+H]^+^ [M−H]^−^
M_hep_1	Hydroxylation (indole) + *O*-Glucuronidation	C_17_H_22_N_2_O_7_	3.06	367.1501 (0.39) 365.1353 (−0.34)	5.2 × 10^5^ 3.6 × 10^5^
M_hep_2	Hydroxylation (indole)	C_11_H_14_N_2_O	4.65	191.1180 (0.42) ND	4.5 × 10^6^ ND
M_hep_3	Hydroxylation (indole) + *O*-Sulfation	C_11_H_14_N_2_O_4_S	5.40	271.0747 (−0.2) 269.0599 (−0.93)	4.1 × 10^5^ 1.6 × 10^6^
M_hep_4	Hydroxylation (indole)	C_11_H_14_N_2_O	6.15	191.1180 (0.63) ND	1.2 × 10^5^ ND
M_hep_5	Hydroxylation (indole) + *O*-Glucuronidation	C_17_H_22_N_2_O_7_	6.35	367.1502 (0.69) 365.1353 (−0.34)	1.6 × 10^5^ 1.3 × 10^5^
M_hep_6	Hydroxylation (indole) + *O*-Sulfation	C_11_H_14_N_2_O_4_S	7.48	271.0748 (0.28) 269.0600 (−0.56)	9.1 × 10^5^ 3.1 × 10^6^
α-MT (Parent)	No transformation	C_11_H_14_N_2_	9.12	175.1231 (0.54) ND	4.5 × 10^7^ ND
M_hep_7	Hydroxylation (indole) + *O*-Sulfation + *N*-Acetylation (indole)	C_13_H_16_N_2_O_5_S	9.48	313.0854 (0.58) 311.0707 (−0.05)	1.2 × 10^5^ 6.7 × 10^5^
M_hep_8	Hydroxylation (indole) + *O*-Glucuronidation + *N*-Acetylation (indole)	C_19_H_24_N_2_O_8_	9.62	409.1609 (0.85) 407.1460 (0.02)	2.9 × 10^5^ 5.2 × 10^5^
M_hep_9	*N*-Acetylation (indole)	C_13_H_16_N_2_O	14.07	217.1336 (0.46) ND	2.7 × 10^6^ ND

**Table 2:** Metabolic transformation, elemental composition, retention time (RT), accurate mass of molecular ion in positive- and negative-ionization modes ([M+H]^+^ and [M−H]^−^, respectively) with mass error, and liquid chromatography–high-resolution mass spectrometry peak area of α-MT and metabolites in postmortem urine with and without enzymatic hydrolysis with β-glucuronidase.

ID	Metabolic Transformation	Elemental Composition	RT, min	*m/z* (Δppm): [M+H]^+^ [M−H]^−^	Peak Area [M+H]^+^ [M−H]^−^	
					Non-Hydrolyzed	Hydrolyzed
M_urine_1	Hydroxylation (indole) + *O*-Glucuronidation	C_17_H_22_N_2_O_7_	2.86	367.1500 (0.06) 365.1357 (0.75)	2.4 × 10^8^ 4.5 × 10^7^	ND
M_urine_2	Hydroxylation (indole)	C_11_H_14_N_2_O	4.62	191.1179 (0.05) ND	2.5 × 10^6^ ND	8.4 × 10^7^ ND
M_urine_3	Hydroxylation (indole) + *O*-Glucuronidation	C_17_H_22_N_2_O_7_	4.81	367.1499 (−0.21) ND	2.7 × 10^6^ ND	ND
M_urine_4	Hydroxylation (indole) + *O*-Glucuronidation	C_17_H_22_N_2_O_7_	4.88	367.1498 (−0.48) ND	2.1 × 10^6^ ND	ND
M_urine_5	Hydroxylation (indole) + *O*-Sulfation	C_11_H_14_N_2_O_4_S	5.08	271.0746 (−0.38) 269.0604 (1.00)	4.4 × 10^7^ 9.7 × 10^7^	3.9 × 10^7^ 1.1 × 10^8^
M_urine_6	Hydroxylation (indole) + *O*-Sulfation	C_11_H_14_N_2_O_4_S	5.36	271.0746 (−0.38) 269.0604 (0.92)	3.4 × 10^8^ 5.3 × 10^8^	3.8 × 10^8^ 5.6 × 10^8^
M_urine_7	Hydroxylation (indole) + *O*-Glucuronidation	C_17_H_22_N_2_O_7_	5.98	367.1502 (0.60) 365.1361 (1.80)	1.2 × 10^7^ 5.2 × 10^6^	2.6 × 10^6^ 1.0 × 10^6^
M_urine_8	Hydroxylation (indole)	C_11_H_14_N_2_O	6.11	191.1181 (1.10) ND	8.5 × 10^5^ ND	2.1 × 10^7^ ND
M_urine_9	Hydroxylation (indole) + *O*-Glucuronidation	C_17_H_22_N_2_O_7_	6.28	367.1501 (0.33) 365.1357 (−0.75)	3.4 × 10^7^ 1.0 × 10^7^	ND
M_urine_10	*N*-Glucuronidation (indole)	C_17_H_22_N_2_O_6_	7.06	351.1548 (−0.75) 349.1409 (1.03)	5.2 × 10^6^ 1.6 × 10^6^	5.2 × 10^6^ 1.6 × 10^6^
M_urine_11	*N*-Glucuronidation (indole)	C_17_H_22_N_2_O_6_	7.15	351.1550 (−0.18) 349.1412 (1.20)	6.6 × 10^6^ 2.0 × 10^6^	7.1 × 10^6^ 1.9 × 10^6^
M_urine_12	Hydroxylation (indole) + *O*-Sulfation	C_11_H_14_N_2_O_4_S	7.44	271.0747 (−0.02) 269.0605 (1.29)	8.5 × 10^7^ 2.3×10^8^	8.0 × 10^7^ 2.3 × 10^8^
α-MT (Parent)	No transformation	C_11_H_14_N_2_	8.56	175.1231 (−0.02) ND	3.5 × 10^9^ ND	3.6 × 10^9^ ND
M_urine_13	Hydroxylation (indole) + *O*-Sulfation + *N*-Acetylation (indole)	C_13_H_16_N_2_O_5_S	9.60	313.0853 (0.71) 311.0711 (1.24)	1.8 × 10^7^ 1.0 × 10^8^	1.6 × 10^7^ 9.3 × 10^7^
M_urine_14	Hydroxylation (indole) + *O*-Glucuronidation + *N*-Acetylation (indole)	C_19_H_24_N_2_O_8_	9.61	409.1609 (0.88) 407.1464 (1.00)	4.2 × 10^6^ 4.0 × 10^6^	ND
M_urine_15	*N*-Glucuronidation (alkyl)	C_17_H_22_N_2_O_6_	10.55	351.1551 (0.16) 349.1412 (1.46)	1.5 × 10^7^ 7.3 × 10^6^	1.5 × 10^7^ 9.0 × 10^6^
M_urine_16	*N*-Acetylation (indole) +*N*-Glucuronidation	C_19_H_24_N_2_O_7_	12.98	393.1661 (1.15) 391.1517 (1.68)	3.0 × 10^7^ 4.4 × 10^7^	3.2 × 10^7^ 4.4 × 10^7^
M_urine_17	*N*-Acetylation (indole)	C_13_H_16_N_2_O	14.08	217.1336 (0.28) ND	5.8 × 10^6^ ND	5.8 × 10^6^ ND

**Table 3:** Metabolic transformation, elemental composition, retention time (RT), accurate mass of molecular ion in positive- and negative-ionization modes ([M+H]^+^ and [M−H]^−^, respectively) with mass error, and liquid chromatography–high-resolution mass spectrometry peak area of α-MT and metabolites in postmortem blood.

ID	Metabolic Transformation	Elemental Composition	RT, min	*m/z* (Δppm): [M+H]^+^ [M−H]^−^	Peak Area [M+H]^+^ [M−H]^−^
M_blood_1	Hydroxylation (indole) + *O*-Sulfation	C_11_H_14_N_2_O_4_S	5.11	271.0750 (1.09) 269.0602 (0.18)	8.5 × 10^5^ 2.3 × 10^6^
M_blood_2	Hydroxylation (indole) + *O*-Sulfation	C_11_H_14_N_2_O_4_S	5.38	271.0750 (1.09) 269.0603 (0.55)	9.2 × 10^6^ 3.1 × 10^7^
M_blood_3	Hydroxylation (indole) + *O*-Sulfation	C_11_H_14_N_2_O_4_S	7.47	271.0750 (1.09) 269.0603 (0.55)	3.5 × 10^6^ 1.3 × 10^7^
α-MT (Parent)	No transformation	C_11_H_14_N_2_	9.04	175.1233 (2.08) ND	1.0 × 10^8^ ND
M_blood_4	Hydroxylation (indole) + *O*-Sulfation + *N*-Acetylation (indole)	C_13_H_16_N_2_O_5_S	9.52	313.0857 (1.38) 311.0711 (1.24)	5.9 × 10^5^ 3.6 × 10^6^
M_blood_5	*N*-Acetylation (indole)	C_13_H_16_N_2_O	14.07	217.1338 (1.20) ND	2.1 × 10^6^ ND

**Table 4:** Metabolic transformation, elemental composition, retention time (RT) in urine, and theoretical accurate mass of molecular ion in positive- and negative-ionization modes ([M+H]^+^ and [M-H]^−^, respectively) of α-MT and metabolites in hepatocyte incubations and postmortem samples. We suggest M_A_, M_F_, and M_L_ as biomarkers of α-MT use in non-hydrolyzed urine, α-MT and M_B_ in hydrolyzed urine, and α-MT, M_F_, M_L_, and M_Q_ in blood.

ID	ID in Samples	Metabolic Transformation	Elemental Composition	Theoretical *m/z*: [M+H]^+^ [M−H]^−^	RT, min
**M_A_**	**=M_hep_1** **=M_urine_1**	**Hydroxylation (indole)** **+ *O*-Glucuronidation**	**C_17_H_22_N_2_O_7_**	**367.1500** **365.1354**	**2.86**
**M_B_**	**=M_hep_2** **=M_urine_2**	**Hydroxylation (indole)**	**C_11_H_14_N_2_O**	**191.1179** **189.1033**	**4.62**
M_C_	=M_urine_3	Hydroxylation (indole) + *O*-Glucuronidation	C_17_H_22_N_2_O_7_	367.1500 365.1354	4.81
M_D_	=M_urine_4	Hydroxylation (indole) + *O*-Glucuronidation	C_17_H_22_N_2_O_7_	367.1500 365.1354	4.88
M_E_	=M_urine_5 =M_blood_1	Hydroxylation (indole) + *O*-Sulfation	C_11_H_14_N_2_O_4_S	271.0747 269.0602	5.08
**M_F_**	**=M_hep_3** **=M_urine_6** **=M_blood_2**	**Hydroxylation (indole)** **+ *O*-Sulfation**	**C_11_H_14_N_2_O_4_S**	**271.0747** **269.0602**	**5.36**
M_G_	=M_urine_7	Hydroxylation (indole) + *O*-Glucuronidation	C_17_H_22_N_2_O_7_	367.1500 365.1354	5.98
M_H_	=M_hep_4 =M_urine_8	Hydroxylation (indole)	C_11_H_14_N_2_O	191.1179 189.1033	6.11
M_I_	=M_hep_5 =M_urine_9	Hydroxylation (indole) + *O*-Glucuronidation	C_17_H_22_N_2_O_7_	367.1500 365.1354	6.28
M_J_	=M_urine_10	*N*-Glucuronidation (indole)	C_17_H_22_N_2_O_6_	351.1551 349.1405	7.06
M_K_	=M_urine_11	*N*-Glucuronidation (indole)	C_17_H_22_N_2_O_6_	351.1551 349.1405	7.15
**M_L_**	**=M_hep_6** **=M_urine_12** **=M_blood_3**	**Hydroxylation (indole)** **+ *O*-Sulfation**	**C_11_H_14_N_2_O_4_S**	**271.0747** **269.0602**	**7.44**
**α-MT** **(Parent)**	**NA**	**No transformation**	**C_11_H_14_N_2_**	**175.1230** **173.1084**	**8.56**
M_M_	=M_hep_7 =M_urine_13 =M_blood_4	Hydroxylation (indole) + *O*-Sulfation + *N*-Acetylation (indole)	C_13_H_16_N_2_O_5_S	313.0853 311.0707	9.60
M_N_	=M_hep_8 =M_urine_14	Hydroxylation (indole) + *O*-Glucuronidation + *N*-Acetylation (indole)	C_19_H_24_N_2_O_8_	409.1605 407.1460	9.61
M_O_	=M_urine_15	*N*-Glucuronidation (alkyl)	C_17_H_22_N_2_O_6_	351.1551 349.1405	10.55
M_P_	=M_urine_16	*N*-Acetylation (indole) +*N*-Glucuronidation	C_19_H_24_N_2_O_7_	393.1656 391.1511	12.98
**M_Q_**	**=M_hep_9** **=M_urine_17** **=M_blood_5**	***N*-Acetylation (indole)**	**C_13_H_16_N_2_O**	**217.1335** **215.1190**	**14.08**

## Data Availability

Raw data were generated at the Department of Biomedical Sciences and Public Health, Marche Polytechnic University. Derived data supporting the findings of this study are available from the corresponding author on request.

## Supplementary Materials

The following supporting information can be downloaded at https://www.mdpi.com/article/10.3390/metabo13010092/s1, Figure S1: HRMS/MS fragmentation of α-MT and metabolites, Table S1: Inclusion lists for LC-HRMS/MS analysis, Table S2: α-MT putative metabolites predicted with BioTransformer freeware.
