# Supplementary material for: α-Methyltryptamine (α-MT) Metabolite Profiling in Human Hepatocyte Incubations and Postmortem Urine and Blood
Source: Metabolites. 2023 Jan 6;13(1):92. doi: 10.3390/metabo13010092 (PMC9866742; doi:10.3390/metabo13010092)
Supplement: Supplementary file 1 [file metabolites-13-00092-s001.zip › metabolites-2107762-supplementary Table S1.pdf]

Table S1. α-MT putative metabolites predicted with BioTransformer freeware. MW, molecular weight; SMILES, simplified molecular input line entry specification.

ID	Transformation	SMILES	Elemental composition	MW (Da)
P1	Hydroxylation (alkyl)	CC(C(C1=CNC2=CC=CC=C21)O)N	$C_{11}H_{14}N_2O$	190.1
P2	Hydroxylation (indole)	CC(CC1=CNC2=CC(=CC=C21)O)N	$C_{11}H_{14}N_2O$	190.1
P3	Hydroxylation (indole)	CC(CC1=CNC2=CC=C(C=C21)O)N	$C_{11}H_{14}N_2O$	190.1
P4	Hydroxylation (indole)	CC(CC1=CNC2=C(C=CC=C21)O)N	$C_{11}H_{14}N_2O$	190.1
P5	Hydroxylation (indole)	CC(CC1=CNC2=CC=CC(=C21)O)N	$C_{11}H_{14}N_2O$	190.1
P6	Hydroxylation (alkyl)	C(C(CC1=CNC2=CC=CC=C21)N)O	$C_{11}H_{14}N_2O$	190.1
P7	Terminal desaturation	C=C(CC1=CNC2=CC=CC=C21)N	$C_{11}H_{12}N_2$	172.1
P8	Oxidative deamination	CC(CC1=CNC2=CC=CC=C12)=O	$C_{11}H_{11}NO$	173.1
P9	*N*-Oxidation (indole)	CC(CC1=C[NH+](C2=CC=CC=C12)[O-])N	$C_{11}H_{14}N_2O$	190.1
P10	*N*-Oxidation (alkyl)	CC(CC1=CNC2=CC=CC=C12)[NH2+][O-]	$C_{11}H_{14}N_2O$	190.1
P11	Hydroxylation (alkyl) + *O*-Glucuronidation	CC(C(C1=CNC2=CC=CC=C21)OC3OC(C(O)=O)C(C(C3O)O)O)N	$C_{17}H_{22}N_2O_7$	366.1
P12	Hydroxylation (alkyl) + *O*-Sulfation	CC(C(C1=CNC2=CC=CC=C21)OS(O)(=O)=O)N	$C_{11}H_{14}N_2O_4S$	270.1
P13	Hydroxylation (alkyl) + Deaturation to ketone	CC(C(C1=CNC2=CC=CC=C21)=O)N	$C_{11}H_{12}N_2O$	188.1
P14	Hydroxylation (alkyl) + Hydroxylation (indole)	CC(C(C1=CNC2=CC(=CC=C21)O)O)N	$C_{11}H_{14}N_2O_2$	206.1
P15	Hydroxylation (alkyl) + Hydroxylation (indole)	CC(C(C1=CNC2=CC=C(C=C21)O)O)N	$C_{11}H_{14}N_2O_2$	206.1
P16	Hydroxylation (alkyl) + Hydroxylation (indole)	CC(C(C1=CNC2=C(C=CC=C21)O)O)N	$C_{11}H_{14}N_2O_2$	206.1
P17	Hydroxylation (alkyl) + Hydroxylation (indole)	CC(C(C1=CNC2=CC=CC(=C21)O)O)N	$C_{11}H_{14}N_2O_2$	206.1
P18	Hydroxylation (alkyl) + Hydroxylation (alkyl)	C(C(C(C1=CNC2=CC=CC=C21)O)N)O	$C_{11}H_{14}N_2O_2$	206.1
P19	Hydroxylation (alkyl) + Terminal desaturation	C=C(C(C1=CNC2=CC=CC=C21)O)N	$C_{11}H_{12}N_2O$	188.1
P20	Hydroxylation (alkyl) + Hydroxylation (alkyl)	CC(C(C1=CNC2=CC=CC=C12)O)(N)O	$C_{11}H_{14}N_2O_2$	206.1
P21	Hydroxylation (alkyl) + *N*-Oxidation (alkyl)	CC(C(C1=CNC2=CC=CC=C12)O)NO	$C_{11}H_{14}N_2O_2$	206.1
P22	Hydroxylation (alkyl) + *N*-Oxidation (indole)	CC(C(C1=C[NH+](C2=CC=CC=C12)[O-])O)N	$C_{11}H_{14}N_2O_2$	206.1
P23	Hydroxylation (indole) + *O*-Glucuronidation	CC(CC1=CNC2=CC(=CC=C21)OC3OC(C(O)C(O)C3O)C(O)=O)N	$C_{17}H_{22}N_2O_7$	366.1
P24	Hydroxylation (indole) + Hydroxylation (indole)	CC(CC1=CNC2=CC(=C(C=C21)O)O)N	$C_{11}H_{14}N_2O_2$	206.1
P25	Hydroxylation (indole) + Hydroxylation (indole)	CC(CC1=CNC2=CC(=CC(=C21)O)O)N	$C_{11}H_{14}N_2O_2$	206.1
P26	Hydroxylation (indole) + Hydroxylation (alkyl)	C(C(CC1=CNC2=CC(=CC=C21)O)N)O	$C_{11}H_{14}N_2O_2$	206.1
P27	Hydroxylation (indole) + Terminal desaturation	C=C(CC1=CNC2=CC(=CC=C21)O)N	$C_{11}H_{12}N_2O$	188.1
P28	Hydroxylation (indole) + *N*-Oxidation (alkyl)	CC(CC1=CNC2=CC(=CC=C12)O)NO	$C_{11}H_{14}N_2O_2$	206.1
P29	Hydroxylation (indole) + Hydroxylation (alkyl)	CC(CC1=CNC2=CC(=CC=C12)O)(N)O	$C_{11}H_{14}N_2O_2$	206.1
P30	Hydroxylation (indole) + *N*-Oxidation (alkyl)	CC(CC1=CNC2=CC(=CC=C12)O)[NH2+][O-]	$C_{11}H_{14}N_2O_2$	206.1
P31	Hydroxylation (indole) + *O*-Glucuronidation	CC(CC1=CNC2=CC=C(C=C21)OC3OC(C(O)C(O)C3O)C(O)=O)N	$C_{17}H_{22}N_2O_7$	366.1
P32	Hydroxylation (indole) + Hydroxylation (indole)	CC(CC1=CNC2=C(C=C(C=C21)O)O)N	$C_{11}H_{14}N_2O_2$	206.1
P33	Hydroxylation (indole) + Hydroxylation (indole)	CC(CC1=CNC2=CC=C(C(=C21)O)O)N	$C_{11}H_{14}N_2O_2$	206.1
P34	Hydroxylation (indole) + Hydroxylation (alkyl)	C(C(CC1=CNC2=CC=C(C=C21)O)N)O	$C_{11}H_{14}N_2O_2$	206.1
P35	Hydroxylation (indole) + Terminal desaturation	C=C(CC1=CNC2=CC=C(C=C21)O)N	$C_{11}H_{12}N_2O$	188.1
P36	Hydroxylation (indole) + *N*-Oxidation (alkyl)	CC(CC1=CNC2=CC=C(C=C12)O)NO	$C_{11}H_{14}N_2O_2$	206.1
P37	Hydroxylation (indole) + Hydroxylation (alkyl)	CC(CC1=CNC2=CC=C(C=C12)O)(N)O	$C_{11}H_{14}N_2O_2$	206.1
P38	Hydroxylation (indole) + *N*-Oxidation (alkyl)	CC(CC1=CNC2=CC=C(C=C12)O)[NH2+][O-]	$C_{11}H_{14}N_2O_2$	206.1
P39	Hydroxylation (indole) + *O*-Glucuronidation	CC(CC1=CNC2=C(C=CC=C21)OC3OC(C(O)C(O)C3O)C(O)=O)N	$C_{17}H_{22}N_2O_7$	366.1
P40	Hydroxylation (indole) + Hydroxylation (indole)	CC(CC1=CNC2=C(C(=CC=C21)O)O)N	$C_{11}H_{14}N_2O_2$	206.1
P41	Hydroxylation (indole) + Hydroxylation (indole)	CC(CC1=CNC2=C(C=C(C=C21)O)O)N	$C_{11}H_{14}N_2O_2$	206.1
P42	Hydroxylation (indole) + Hydroxylation (alkyl)	C(C(CC1=CNC2=C(C=CC=C21)O)N)O	$C_{11}H_{14}N_2O_2$	206.1
P43	Hydroxylation (indole) + Terminal desaturation	C=C(CC1=CNC2=C(C=CC=C21)O)N	$C_{11}H_{12}N_2O$	188.1
P44	Hydroxylation (indole) + *N*-Oxidation (alkyl)	CC(CC1=CNC2=C(C=CC=C12)O)NO	$C_{11}H_{14}N_2O_2$	206.1
P45	Hydroxylation (indole) + *N*-Oxidation (indole)	CC(CC1=C[NH+](C2=C(C=CC=C12)O)[O-])N	$C_{11}H_{14}N_2O_2$	206.1
P46	Hydroxylation (indole) + Hydroxylation (alkyl)	CC(CC1=CNC2=C(C=CC=C12)O)(N)O	$C_{11}H_{14}N_2O_2$	206.1
P47	Hydroxylation (indole) + *N*-Oxidation (alkyl)	CC(CC1=CNC2=C(C=CC=C12)O)[NH2+][O-]	$C_{11}H_{14}N_2O_2$	206.1
P48	Hydroxylation (indole) + *O*-Glucuronidation	CC(CC1=CNC2=CC=CC(=C21)OC3OC(C(O)C(O)C3O)C(O)=O)N	$C_{17}H_{22}N_2O_7$	366.1
P49	Hydroxylation (indole) + Hydroxylation (alkyl)	C(C(CC1=CNC2=CC=CC(=C21)O)N)O	$C_{11}H_{14}N_2O_2$	206.1
P50	Hydroxylation (indole) + Terminal desaturation	C=C(CC1=CNC2=CC=CC(=C21)O)N	$C_{11}H_{12}N_2O$	188.1
P51	Hydroxylation (indole) + *N*-Oxidation (alkyl)	CC(CC1=CNC2=CC=CC(=C12)O)NO	$C_{11}H_{14}N_2O_2$	206.1
P52	Hydroxylation (indole) + Hydroxylation (alkyl)	CC(CC1=CNC2=CC=CC(=C12)O)(N)O	$C_{11}H_{14}N_2O_2$	206.1
P53	Hydroxylation (indole) + *N*-Oxidation (alkyl)	CC(CC1=CNC2=CC=CC(=C12)O)[NH2+][O-]	$C_{11}H_{14}N_2O_2$	206.1
P54	Hydroxylation (alkyl) + *O*-Glucuronidation	C(C(CC1=CNC2=CC=CC=C21)N)OC3OC(C(O)=O)C(C(C3O)O)O	$C_{17}H_{22}N_2O_7$	366.1
P55	Hydroxylation (alkyl) + *O*-Sulfation	C(C(CC1=CNC2=CC=CC=C21)N)OS(O)(=O)=O	$C_{11}H_{14}N_2O_4S$	270.1
P56	Hydroxylation (alkyl) + Desaturation to aldehyde	C(C(CC1=CNC2=CC=CC=C21)N)=O	$C_{11}H_{12}N_2O$	188.1
P57	Hydroxylation (alkyl) + Hydroxylation (alkyl)	C(O)C(CC1=CNC2=CC=CC=C12)(N)O	$C_{11}H_{14}N_2O_2$	206.1
P58	Hydroxylation (alkyl) + *N*-Oxidation (alkyl)	C(C(CC1=CNC2=CC=CC=C12)NO)O	$C_{11}H_{14}N_2O_2$	206.1
P59	Terminal desaturation + *N*-Oxidation (indole)	C=C(CC1=C[NH+](C2=CC=CC=C12)[O-])N	$C_{11}H_{12}N_2O$	188.1
P60	Terminal desaturation + *N*-Oxidation (alkyl)	C=C(CC1=CNC2=CC=CC=C12)NO	$C_{11}H_{12}N_2O$	188.1
P61	Oxidative deamination + Hydroxylation (alkyl)	CC(C(C1=CNC2=CC=CC=C12)O)=O	$C_{11}H_{11}NO_2$	189.1
P62	Oxidative deamination + Hydroxylation (indole)	CC(CC1=CNC2=CC(=CC=C12)O)=O	$C_{11}H_{11}NO_2$	189.1
P63	Oxidative deamination + Hydroxylation (indole)	CC(CC1=CNC2=CC=C(C=C12)O)=O	$C_{11}H_{11}NO_2$	189.1
P64	Oxidative deamination + Hydroxylation (indole)	CC(CC1=CNC2=C(C=CC=C12)O)=O	$C_{11}H_{11}NO_2$	189.1
P65	Oxidative deamination + Hydroxylation (indole)	CC(CC1=CNC2=CC=CC(=C12)O)=O	$C_{11}H_{11}NO_2$	189.1
P66	Oxidative deamination + Hydroxylation (alkyl)	C(C(CC1=CNC2=CC=CC=C12)=O)O	$C_{11}H_{11}NO_2$	189.1

P67	Oxidative deamination + Hydroxylation (alkyl)	CC(C(C1=CNC2=CC=CC=C12)O)=O	$C_{11}H_{11}NO_2$	189.1
P68	Oxidative deamination + Ketoreduction	CC(CC1=CNC2=CC=CC=C12)O	$C_{11}H_{13}NO$	175.1
P69	Oxidative deamination + *N*-Oxidation	CC(CC1=C[NH+](C2=CC=CC=C12)[O-])=O	$C_{11}H_{11}NO_2$	189.1
P70	*N*-Oxidation (indole) + Hydroxylation (indole)	CC(CC1=C[NH+](C2=CC(=CC=C12)O)[O-])N	$C_{11}H_{14}N_2O_2$	206.1
P71	*N*-Oxidation (indole) + Hydroxylation (indole)	CC(CC1=C[NH+](C2=CC=C(C=C12)O)[O-])N	$C_{11}H_{14}N_2O_2$	206.1
P72	*N*-Oxidation (indole) + Hydroxylation (indole)	CC(CC1=C[NH+](C2=CC=CC(=C12)O)[O-])N	$C_{11}H_{14}N_2O_2$	206.1
P73	*N*-Oxidation (indole) + Hydroxylation (alkyl)	C(C(CC1=C[NH+](C2=CC=CC=C12)[O-])N)O	$C_{11}H_{14}N_2O_2$	206.1
P74	*N*-Oxidation (alkyl) + Hydroxylation (alkyl)	CC(C(C1=CNC2=CC=CC=C12)O)[NH2+][O-]	$C_{11}H_{14}N_2O_2$	206.1
P75	*N*-Oxidation (alkyl) + Hydroxylation (alkyl)	C(C(CC1=CNC2=CC=CC=C12)[NH2+][O-])O	$C_{11}H_{14}N_2O_2$	206.1
P76	*N*-Oxidation (alkyl) + Terminal desaturation	C=C(CC1=CNC2=CC=CC=C12)[NH2+][O-]	$C_{11}H_{12}N_2O$	188.1
P77	*N*-Oxidation (alkyl) + Hydroxylation (alkyl)	CC(CC1=CNC2=CC=CC=C12)([NH2+][O-])O	$C_{11}H_{14}N_2O_2$	206.1
P78	*N*-Oxidation (alkyl) + *N*-Oxidation (alkyl)	CC(CC1=CNC2=CC=CC=C12)[NH+]([O-])O	$C_{11}H_{14}N_2O_2$	206.1
P79	*N*-Oxidation (alkyl) + *N*-Oxidation (indole)	CC(CC1=C[NH+](C2=CC=CC=C12)[O-])[NH2+][O-]	$C_{11}H_{14}N_2O_2$	206.1
