# Supplementary material for: α-Methyltryptamine (α-MT) Metabolite Profiling in Human Hepatocyte Incubations and Postmortem Urine and Blood
Source: Metabolites. 2023 Jan 6;13(1):92. doi: 10.3390/metabo13010092 (PMC9866742; doi:10.3390/metabo13010092)
Supplement: Supplementary file 1 [file metabolites-13-00092-s001.zip › metabolites-2107762-supplementary Table S2.pdf]

Table S2. Inclusion lists for liquid chromatography-high-resolution tandem mass spectrometry analysis.

**Composition change**	**Elemental composition**	**$[M+H]^+$, *m/z***	**$[M-H]^-$, *m/z***	**Reference transformation**
-	$C_{11}H_{14}N_2$	175.1229	173.1084	None (parent, αMT)
+O	$C_{11}H_{14}N_2O$	191.1178	189.1033	Hydroxylation
-2H	$C_{11}H_{12}N_2$	173.1073	171.0927	Desaturation
-3H -N +O	$C_{11}H_{11}NO$	174.0913	172.0767	Deamination to ketone
+6C +8H +7O	$C_{17}H_{22}N_2O_7$	367.1499	365.1354	Hydroxylation + Glucuronidation
+4O +S	$C_{11}H_{14}N_2O_4S$	271.0747	269.0601	Hydroxylation + Sulfation
-2H +O	$C_{11}H_{12}N_2O$	189.1022	187.0876	Ketone formation
+2O	$C_{11}H_{14}N_2O_2$	207.1128	205.0982	Di-hydroxylation
-3H -N +2O	$C_{11}H_{11}NO_2$	190.0862	188.0717	Deamination to ketone + Hydroxylation
-H -N +O	$C_{11}H_{13}NO$	176.1069	174.0924	Deamination to alcohol
+6C +8H +6O	$C_{17}H_{22}N_2O_6$	351.1551	349.1405	Glucuronidation
+3O +S	$C_{11}H_{14}N_2O_3S$	255.0798	253.0652	Sulfation
+2H +2O	$C_{11}H_{16}N_2O_2$	209.1285	207.1139	Dihydrodiol formation
+10C +15H +3N +6O +S	$C_{21}H_{29}N_5O_6S$	480.1911	478.1766	Epoxidation + Glutathionylation
+5C +8H +2N +3O +S	$C_{16}H_{22}N_4O_3S$	351.1485	349.1340	Epoxidation + Cys-Gly conjugation
+3C +5H +N +2O +S	$C_{14}H_{19}N_3O_2S$	294.1271	292.1125	Epoxidation + Cys conjugation
+10C +17H +3N +7O +S	$C_{21}H_{31}N_5O_7S$	498.2017	496.1871	Dihydrodiol formation + Glutathionylation
+5C +10H +2N +4O +S	$C_{16}H_{24}N_4O_4S$	369.1591	367.1445	Dihydrodiol formation + Cys-Gly conjugation
+3C +7H +N +3O +S	$C_{14}H_{21}N_3O_3S$	312.1376	310.1231	Dihydrodiol formation + Cys conjugation
